# Metagenome-assembled genomes recovered from the gut microbiomes of simian immunodeficiency virus-infected rhesus macaques

**DOI:** 10.1128/mra.00428-26

**Published:** 2026-06-22

**Authors:** Eric Q. Pham, Christopher A. Gaulke, Jonathan A. Eisen, Satya Dandekar

**Affiliations:** 1 Department of Medical Microbiology and Immunology, University of California, Davis 8789, Davis, California, USA; 2 Department of Pathobiology, University of Illinois Urbana-Champaign 14589, Urbana, Illinois, USA; 3 National Center for Supercomputing Applications, University of Illinois Urbana-Champaign 14589, Urbana, Illinois, USA; 4 Department of Evolution and Ecology, University of California, Davis 8789, Davis, California, USA; 5 Genome Center, University of California, Davis, Davis, California, USA; 6 California National Primate Research Center, University of California, Davis 8789, Davis, California, USA; Montana State University, Bozeman, Montana, USA

**Keywords:** metagenome-assembled genomes, gut microbiome, rhesus macaque

## Abstract

Rhesus macaques are widely used model organisms for studying human biology, yet relatively few metagenome-assembled genomes (MAGs) are available from their microbiome. Here, we report 159 MAGs recovered from simian immunodeficiency virus-infected macaques, including those treated either with antiretroviral therapy or 10-hydroxystearic acid.

## ANNOUNCEMENT

Non-human primates (NHPs) are essential model organisms for preclinical and translational research due to their physiological, anatomical, genetic, and behavioral similarities to humans (1, 2). Among these, rhesus macaques (*Macaca mulatta*) account for the majority of NHP studies for research on infectious diseases, neurological disorders, development, and aging (1, 2). However, genomic resources characterizing the rhesus macaque gut microbiome remain limited, with relatively few publicly available metagenome-assembled genome (MAG) data sets (3, 4). To address this gap, we generated 159 gut microbiome-associated MAGs from rhesus macaques that substantially expand genomic resources for this model system.

Fecal samples (*n* = 28) were collected using rectal swabs from nine simian immunodeficiency virus (SIV)-infected rhesus macaques, an established NHP model of HIV/AIDS. Rhesus macaques at the California National Primate Research Center were infected with SIV (1,000 tissue culture infectious dose 50% [TCID_50_] SIVmac251) and received either antiretroviral therapy (5.1 mg/kg tenofovir disoproxil fumarate, 40 mg/kg emtricitabine, and 2.5 mg/kg dolutegravir; *n* = 3) or supplementation of 10-hydroxystearic acid (*n* = 3) or no treatment (*n* = 3). DNA was isolated using the DNeasy PowerSoil Pro Kit (Qiagen). Samples were incubated at 65°C for 10 min before bead beating. Libraries were prepared using the Watchmaker DNA Library Prep Kit with enzymatic fragmentation, and fragments smaller than 400 bp long were excluded by using solid-phase reversible immobilization beads. Libraries were sequenced on an Illumina NovaX platform, generating paired-end 150 bp reads (UC Davis DNA Technologies and Expression Analysis Core). All procedures were performed in accordance with the Public Health Services Policy, American Veterinary Medical Association guidelines, and protocols approved by the Institutional Animal Care and Use Committee at the University of California, Davis.

**Fig 1:**
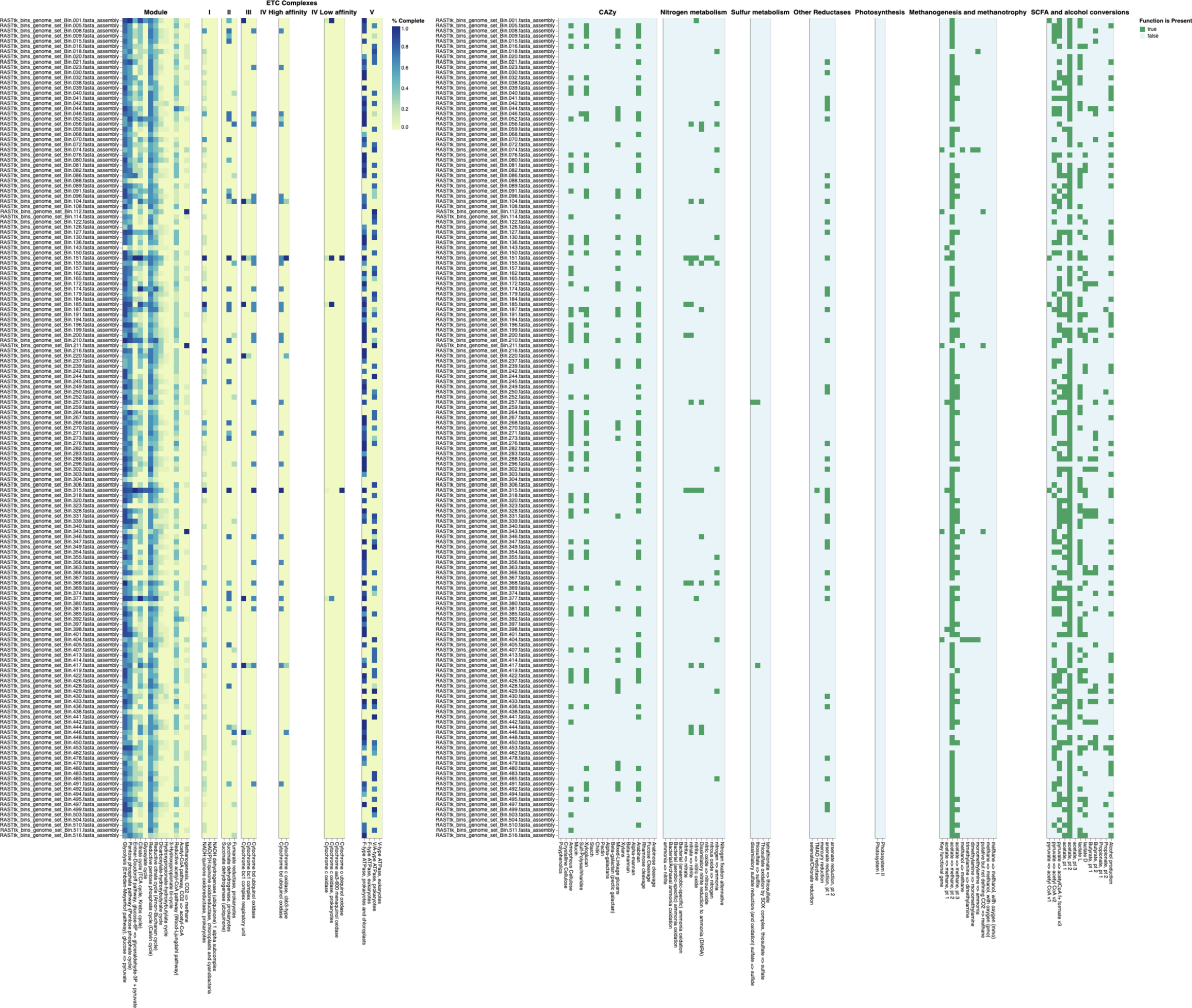
Heatmaps of DRAMv0.1.2 annotations of MAGs showing a large diversity of potential metabolism in the rhesus macaque gut microbiome. Many organisms contain enzymes related to short-chain fatty acid (SCFA) and alcohol metabolism, while some maintain carbohydrate-active enzymes (CAZymes), nitrogen, and methane metabolism, and few have enzymes related to sulfur metabolism.

Raw reads were trimmed and quality-filtered with cutadapt v2.6 (5) using parameters --minimum-length=50 --max-n=1 q 30 -u 15 -U 15. Host-derived reads were removed with Bowtie2 v2.5.1 (6) against the *M. mulatta* reference genome (Mmul_10 genome; GCA_003339765.3). Downstream analyses were performed in KBase (7). All samples, totaling 3,209,036,744 reads, were co-assembled using MEGAHIT v1.2.9 (8) with the meta-large parameter and produced 748,374 contigs (N50 = 13,070 bp). Binning was executed with MaxBin2 v2.2.4 (9) (minimum contig length = 6,000) and MetaBAT2 v1.7 (10) (minimum contig length = 5,000), generating 852 and 999 bins, respectively, followed by refinement to 520 bins with DAS tool v1.1.2 (11). Genome quality was assessed using CheckM v1.0.18 (12), and 159 high-quality bacterial and archaeal MAGs were filtered (>90% completeness; <5% contamination). Annotation was performed using RASTtk v1.073 (13), and taxonomic classification was assigned with GTDB-Tk v2.3.2 (14). Metabolic annotation was completed using DRAM v0.1.2 (15) (Fig. 1). MAGs were deposited to the National Center for Biotechnology Information (NCBI) and annotated with the Prokaryotic Genome Annotation Pipeline (16, 17).

Of the 159 MAGs, nearly a quarter were assigned to a taxonomic level higher than species (35 to genus, three to family, one to order) (Table 1). The MAGs encompass 19 classes with most belonging to *Clostridia* (75/159; 47%), *Bacilli* (23/159; 14%), or *Bacteroidia* (19/159; 12%). These MAGs expand available genomic references and provide a resource for future studies of host–microbiome interactions in NHP models of infectious disease.

**TABLE 1:** Count of MAGs by class assigned by GTDB-Tk v2.3.2 (14)

Class	MAG count
*Actinomycetia*	2
*Alphaproteobacteria*	4
*Bacilli*	23
*Bacteroidia*	19
*Campylobacteria*	3
*Clostridia*	75
*Coriobacteriia*	3
*Deferribacteres*	1
*Desulfovibrionia*	1
*Elusimicrobia*	2
*Fibrobacteria*	1
*Fusobacteriia*	1
*Gammaproteobacteria*	7
*Methanobacteria*	3
*Negativicutes*	2
*Planctomycetia*	1
*Spirochaetia*	7
*Thermoplasmata*	3
*Verrucomicrobiae*	1

## Data Availability

All reads and MAGs are available from the NCBI Sequence Read Archive (SRA) under Bioproject PRJNA1150326. MAGs are deposited under BioSample accession numbers SAMN56786986–SAMN56787144. Raw reads are deposited under BioSample numbers SAMN43277243–SAMN43277251 and SAMN56814328. Assembly, binning, and annotation files are available at https://zenodo.org/records/19372357.
